# Photobiomodulation Improves Kidney Function and Attenuates Oxidative Stress and Inflammation in a Male Wistar Rat Model of Diabetic Kidney Disease

**DOI:** 10.3390/ijms27156678

**Published:** 2026-07-27

**Authors:** Jessica Paola Garcia Villalba, Eloiza de Oliveira Silva, Juliana Veloso Gusmão Silva, Carla Djamila de Pina Victoria, Maikol Lucas de Camargo Gonçalves, Rildo Aparecido Volpini, Laura Giovanna Fernandes Vattimo, Maria de Fatima Fernandes Vattimo

**Affiliations:** 1 School of Nursing, University of Sao Paulo, Sao Paulo 05403-000, Brazil; eloizaosilva@usp.br (E.d.O.S.); juliana.veloso.silva@usp.br (J.V.G.S.); carlavictoria2001@usp.br (C.D.d.P.V.); maikollcg@gmail.com (M.L.d.C.G.); nephron@usp.br (M.d.F.F.V.); 2 Ribeirao Preto Medical School, University of Sao Paulo, Ribeirão Preto 14049-900, Brazil; rildovolpini@fmrp.usp.br; 3 Institute of Mathematics, Statistics and Computer Science, University of Sao Paulo, Sao Paulo 05508-090, Brazil; lg.vattimo@gmail.com

**Keywords:** photobiomodulation, diabetic kidney disease, oxidative stress, inflammation

## Abstract

Diabetic kidney disease (DKD) is characterized by progressive renal dysfunction driven by oxidative stress, inflammation, and hemodynamic alterations. Photobiomodulation (PBM) has emerged as a potential non-invasive therapeutic strategy targeting these pathways. To evaluate the effects of PBM on kidney function, hemodynamics, oxidative stress, and inflammatory profile in a rat model of DKD. Adult male Wistar rats were randomly assigned to four groups: control (Ct), Ct + PBM, DKD, and DKD + PBM. Diabetes was induced by streptozotocin (60 mg/kg, i.v.). PBM (808 nm, 100 mW) was applied transcutaneously (3 J per point; 30.48 J/cm^2^), bilaterally over the renal region, three times per week for six weeks. Kidney function (inulin clearance, serum creatinine, albuminuria), hemodynamics (mean arterial pressure, renal blood flow, renal vascular resistance), oxidative stress markers (urinary H_2_O_2_, NOx, thiols, Nrf2), and IL-1β levels were evaluated. DKD animals showed impaired kidney function, increased oxidative stress, and elevated inflammatory markers. PBM treatment significantly improved inulin clearance, reduced albuminuria and serum creatinine, increased renal blood flow, and decreased mean arterial pressure and vascular resistance. Oxidative stress markers and IL-1β levels were attenuated, with partial restoration of antioxidant capacity. No significant histological differences were observed. PBM improves kidney function and modulates redox and inflammatory pathways in experimental DKD, supporting its potential as a non-invasive adjunct therapy.

## 1. Introduction

Every nine seconds, one person dies as a result of complications from diabetes mellitus (DM), totaling approximately 3.4 million deaths in 2024. These figures reflect not only the global magnitude of this chronic disease but also the devastating impact of its complications, among which diabetic kidney disease (DKD) stands out as one of the most severe and silent microangiopathic manifestations of DM [1].

DM is a chronic metabolic condition characterized by persistent hyperglycemia, resulting from a deficiency in insulin production or resistance to its action. Its prevalence has been increasing exponentially, and it currently represents one of the greatest public health challenges worldwide [2]. DKD represents the most common cause of progression to end-stage chronic kidney disease (ESKD), requiring renal replacement therapy (RRT), such as dialysis or kidney transplantation.

DKD is initially characterized by the presence of albuminuria, and is often accompanied by systemic arterial hypertension (SAH) and a progressive decline in glomerular filtration rate (GFR) [3,4]. When left uncontrolled, it progresses to glomerulosclerosis, interstitial fibrosis, and ultimately renal failure. Chronic kidney disease (CKD), by definition, involves persistent kidney injury evidenced by proteinuria, histological alterations, or a glomerular filtration rate (GFR) below 60 mL/min/1.73 m^2^ for a period longer than three months [3,4].

Its pathophysiology involves a complex interaction between chronic hyperglycemia, mitochondrial dysfunction, oxidative stress, and inflammation, processes that act synergistically in glomerular and tubulointerstitial injury [5,6].

Despite advances in clinical management, CKD still has no cure, and the available therapies aim only to slow its progression. The worsening of renal function significantly affects patients’ quality of life and represents a high cost for healthcare systems due to recurrent hospitalizations and associated complications. In view of the high prevalence of DKD, its insidious progression, and the socioeconomic impact associated with renal failure, the development of innovative therapeutic strategies that act directly on the disease’s pathophysiological mechanisms has become urgent [5].

Photobiomodulation (PBM) is a non-pharmacological approach that employs light in the red and near-infrared spectra, within a wavelength range of approximately 630–1100 nm, to trigger photochemical changes in cellular structures. This process occurs without significant thermal release, distinguishing PBM from other light-based therapies and classifying it as a non-thermal, non-ionizing modality. Light is applied at specific wavelengths, and the resulting photophysical and photochemical processes may stimulate or inhibit various biological responses depending on the target tissue, irradiance, fluence, pulse frequency, spot size, and treatment regimen [6].

Several hypotheses have been proposed to explain how light interacts with cellular chromophores to trigger these biochemical responses. These include: (1) acceleration of electron transfer along the respiratory chain following chromophore excitation, with local temperature increases capable of modulating enzymatic activity; (2) oxygen reduction in the respiratory chain generating superoxide anion, H_2_O_2_, and hydroxyl radicals, with photoabsorbent molecules, such as porphyrins and flavins, acting as reversible photosensitizers for singlet oxygen production; (3) primary photochemical changes in chromophores triggering downstream biochemical reactions that proceed independently of further light activation (photosignaling); and (4) modulation of cellular homeostasis parameters through oxidation–reduction shifts, contributing to the regulation of multiple cellular functions. Additionally, the absorption of infrared-A (IR-A) radiation may activate mitochondrial retrograde signaling pathways, enabling mitochondria to communicate their functional state to the cell nucleus and thereby influence gene expression and cellular response. These converging mechanistic hypotheses provide a biological rationale for the therapeutic application of PBM across multiple pathological contexts [6,7]. Recent studies have shown that PBM can exert antioxidant, anti-inflammatory, and biostimulatory effects through the modulation of the mitochondrial respiratory chain and nitric oxide (NO) metabolism [5,6,7], which are central factors in the pathophysiology of DKD. Although experimental advances suggest beneficial effects of PBM in different models of tissue injury, evidence regarding its application in models of diabetic nephropathy is still scarce, particularly with respect to the modulation of nitric oxide metabolism and its impact on kidney function [6,8,9].

Therefore, considering the limited evidence on the renal effects of photobiomodulation in experimental models of diabetic nephropathy, further investigation is warranted to elucidate its potential mechanisms of action and therapeutic benefits. Given the pivotal role of oxidative stress, inflammation, and hemodynamic alterations in the pathogenesis of DKD, understanding how PBM modulates these processes may provide new insights into renal protection and disease modulation. Thus, the present study aimed to evaluate the effects of photobiomodulation on kidney function, renal hemodynamics, oxidative and antioxidant profiles, and inflammatory markers in rats with diabetic kidney disease, contributing to a better understanding of its renoprotective potential.

## 2. Results

### 2.1. General Physiological Parameters

Physiological parameters were assessed by weekly measurement of capillary blood glucose, food intake, water intake, and urinary flow.

**Figure 1:**
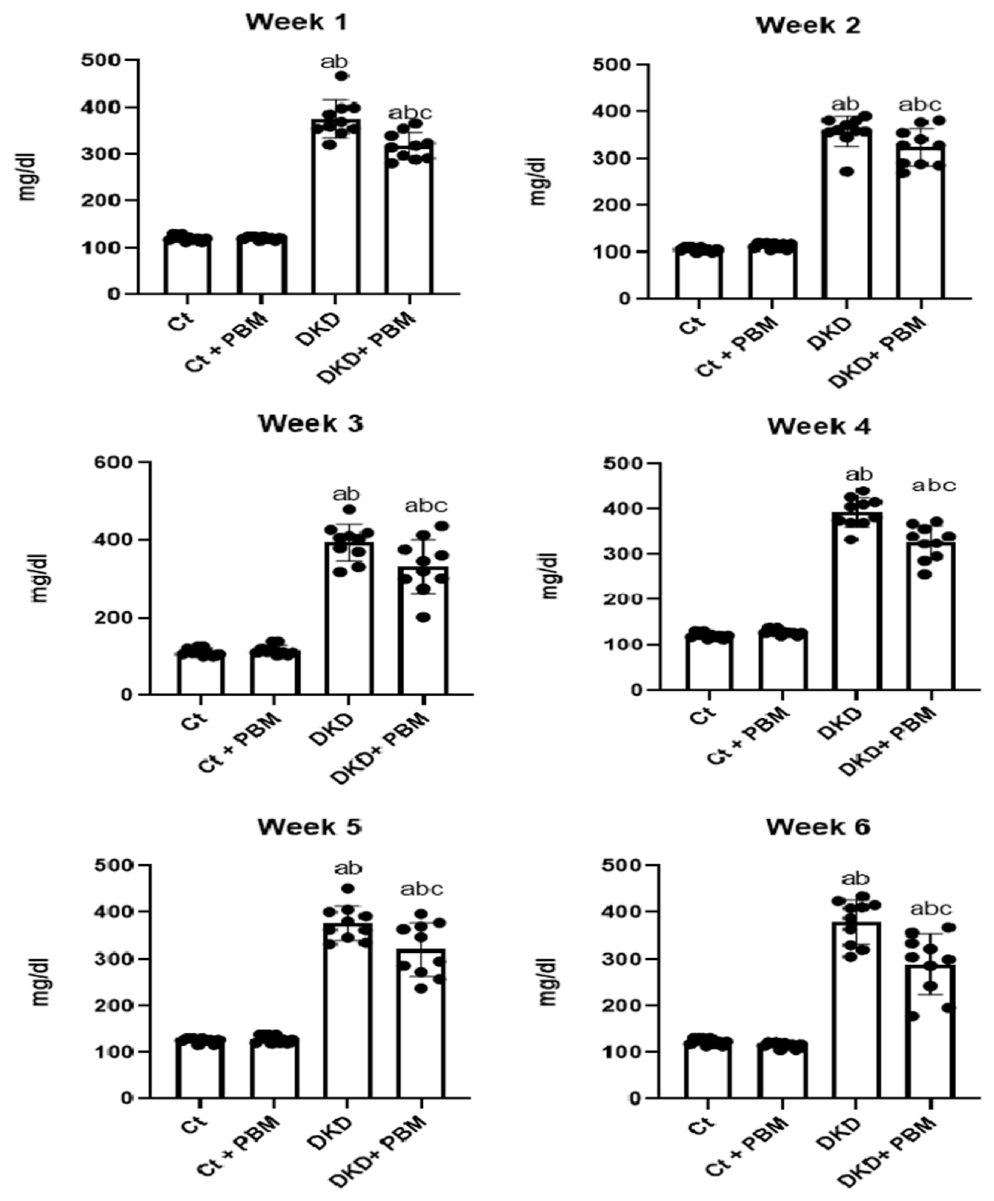
Capillary blood glucose levels during the experimental protocol. Blood glucose (mg/dL) was measured at 48 h after induction and weekly for six weeks in Ct, Ct + PBM, DKD, and DKD + PBM groups. Data are expressed as mean ± SD. Data were analyzed using one-way analysis of variance (ANOVA), followed by Tukey’s multiple comparisons test. No significant difference was observed between Ct and Ct + PBM (mean difference = −9.90; 95% CI [−33.87 to 14.07]; *p* = 0.6844). The DKD and DKD + PBM groups showed significantly higher glycemia compared with Ct (mean difference = −249.5; 95% CI [−273.5 to −225.5]; *p* < 0.0001, and mean difference = −210.7; 95% CI [−234.7 to −186.7]; *p* < 0.0001, respectively) and with Ct + PBM (mean difference = −239.6; 95% CI [−263.6 to −215.6]; *p* < 0.0001, and mean difference = −200.8; 95% CI [−224.8 to −176.8]; *p* < 0.0001, respectively). Notably, the DKD + PBM group showed significantly reduced glycemia compared with the DKD group (mean difference = 38.80; 95% CI [14.83 to 62.77]; *p* = 0.0006), evidencing a hypoglycemic effect of photobiomodulation. Note: a—*p* < 0.05 vs. Ct; b—*p* < 0.05 vs. Ct + PBM; c—*p* < 0.05 vs. DKD.

During the six-week follow-up period, no statistically significant differences in glycemic levels were observed between the Ct and Ct + PBM groups. In contrast, the DKD and DKD + PBM groups showed a significant increase in blood glucose compared to the non-diabetic groups, which persisted throughout all evaluated weeks. Importantly, the DKD + PBM group exhibited a trend toward reduced glycemic levels compared to the DKD group, with significant differences observed from the first week of measurement (Figure 1).

**Figure 2:**
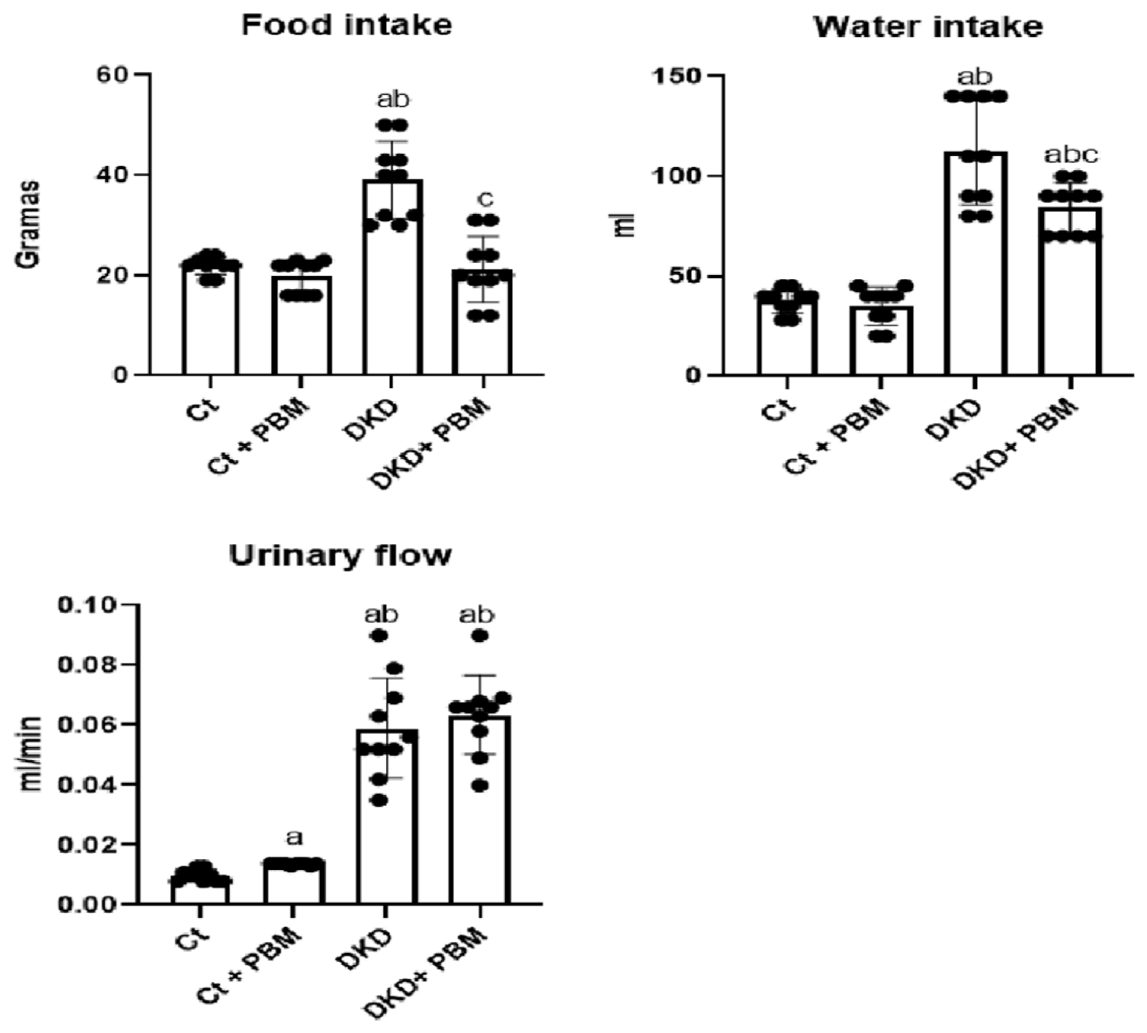
Food, water, and urinary flow evaluation at the end of the experimental protocol. Food intake, water intake, and urinary flow were assessed over a 24 h period at the end of the experimental protocol, with animals individually housed in metabolic cages. Data are expressed as mean ± SD. Statistical analysis was performed using one-way ANOVA, followed by Tukey’s post hoc test. Food intake (g/24 h): DKD differed significantly from Ct and Ct + PBM (*p* < 0.0001); DKD + PBM differed from DKD (mean difference = 17.80; 95% CI [11.29 to 24.31]; *p* < 0.0001), normalizing to levels comparable with controls. Water intake (mL/24 h): DKD differed significantly from Ct and Ct + PBM (*p* < 0.0001); DKD + PBM also differed significantly from both controls (*p* < 0.0001), with a significant reduction compared with DKD (mean difference = 28.00; 95% CI [9.24 to 46.76]; *p* = 0.0016). Urinary flow (mL/min): No significant difference was found between Ct and Ct + PBM (mean difference = −0.0038; 95% CI [−0.0167 to 0.0091]; *p* = 0.8557). The DKD and DKD + PBM groups showed significantly higher urinary flow compared with Ct and Ct + PBM (*p* < 0.0001 for all comparisons), confirming polyuria characteristic of experimental diabetes. No significant difference was found between DKD and DKD + PBM (mean difference = −0.0045; 95% CI [−0.0174 to 0.0084]; *p* = 0.7821). Note: a—*p* < 0.05 vs. Ct; b—*p* < 0.05 vs. Ct + PBM; c—*p* < 0.05 vs. DKD.

No statistically significant differences in food intake were observed between the Ct and Ct + PBM control groups. However, the DKD groups showed a marked increase in food consumption compared with the non-diabetic groups. Furthermore, the DKD + PBM group exhibited a significant reduction in food intake relative to the DKD group similar to the Ct group in this parameter (Figure 2).

With respect to water intake, no significant differences were detected between the control groups. In contrast, both DKD groups displayed a pronounced increase in water consumption compared with controls, consistent with the polydipsia typically associated with diabetes. Importantly, the DKD + PBM group showed a statistically significant reduction in water intake compared with the DKD group (Figure 2).

Regarding urinary flow, the Ct + PBM group exhibited a statistically significant increase compared with the Citrate group. More notably, both DKD groups demonstrated a markedly higher urinary volume than controls, confirming the occurrence of polyuria, a hallmark feature of experimental diabetes mellitus. However, no statistically significant differences were observed between the DKD and DKD + PBM groups (Figure 2).

### 2.2. Renal Hemodynamic Parameters

**Figure 3:**
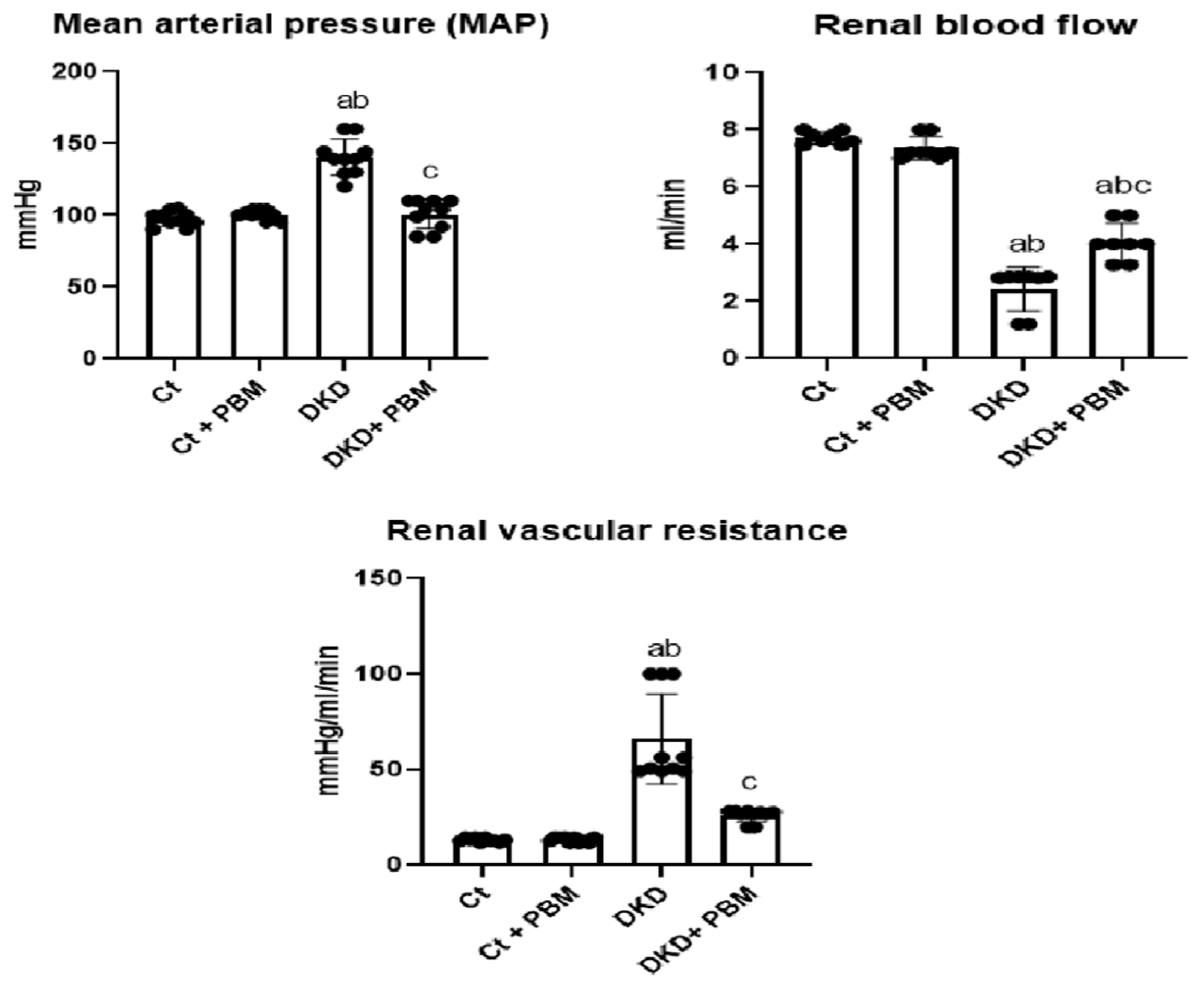
Renal hemodynamic parameters. Mean arterial pressure (MAP, mmHg), renal blood flow (mL/min), and renal vascular resistance (mmHg/mL/min) were evaluated at the end of the experimental protocol in Ct, Ct + PBM, DKD, and DKD + PBM groups. Data are expressed as mean ± SD. Statistical analysis was performed using one-way ANOVA, followed by Tukey’s post hoc test. MAP: DKD differed significantly from Ct and Ct + PBM (*p* < 0.0001); DKD + PBM differed from DKD (mean difference = 39.50; 95% CI [29.00 to 50.00]; *p* < 0.0001), normalizing to levels comparable with controls. Renal blood flow: DKD and DKD + PBM differed significantly from Ct and Ct + PBM (*p* < 0.0001); DKD + PBM differed from DKD (mean difference = −1.638; 95% CI [−2.393 to −0.8823]; *p* < 0.0001), although it remained significantly lower than controls. Renal vascular resistance: DKD differed significantly from Ct and Ct + PBM (*p* < 0.0001); DKD + PBM differed from DKD (mean difference = 40.13; 95% CI [25.74 to 54.51]; *p* < 0.0001), with no significant difference relative to Ct or Ct + PBM. Note: a—*p* < 0.05 vs. Ct; b—*p* < 0.05 vs. Ct + PBM; c—*p* < 0.05 vs. DKD.

In the analysis of renal hemodynamics (Figure 3), no significant differences in mean arterial pressure (MAP) were observed between the control groups. However, animals in the DKD group exhibited a significant increase in MAP compared with controls, consistent with a hypertensive state characteristic of the diabetic experimental model. PBM intervention significantly reduced MAP in the DKD + PBM group compared with the DKD group, suggesting a potential antihypertensive effect.

Regarding renal blood flow, the Ct and Ct + PBM groups presented statistically similar values. In contrast, both DKD groups showed a significant reduction in renal blood flow compared with controls, indicating impaired renal perfusion associated with the diabetic state. Importantly, the DKD + PBM group exhibited a significant increase in renal blood flow compared with the DKD group, suggesting a beneficial effect of PBM on renal hemodynamics, but this remained significantly lower than the control groups.

For renal vascular resistance, the Ct and Ct + PBM groups maintained values within the normal range, with no statistically significant differences between them. In contrast, both the DKD and DKD + PBM groups displayed a significant increase in renal vascular resistance compared with controls, reflecting endothelial dysfunction and vascular tone alterations typical of diabetes. Notably, the DKD + PBM group showed a significant reduction in this parameter compared with the DKD group, reinforcing the modulatory effect of PBM on renal vascular resistance.

### 2.3. Kidney Function Parameters

**Figure 4:**
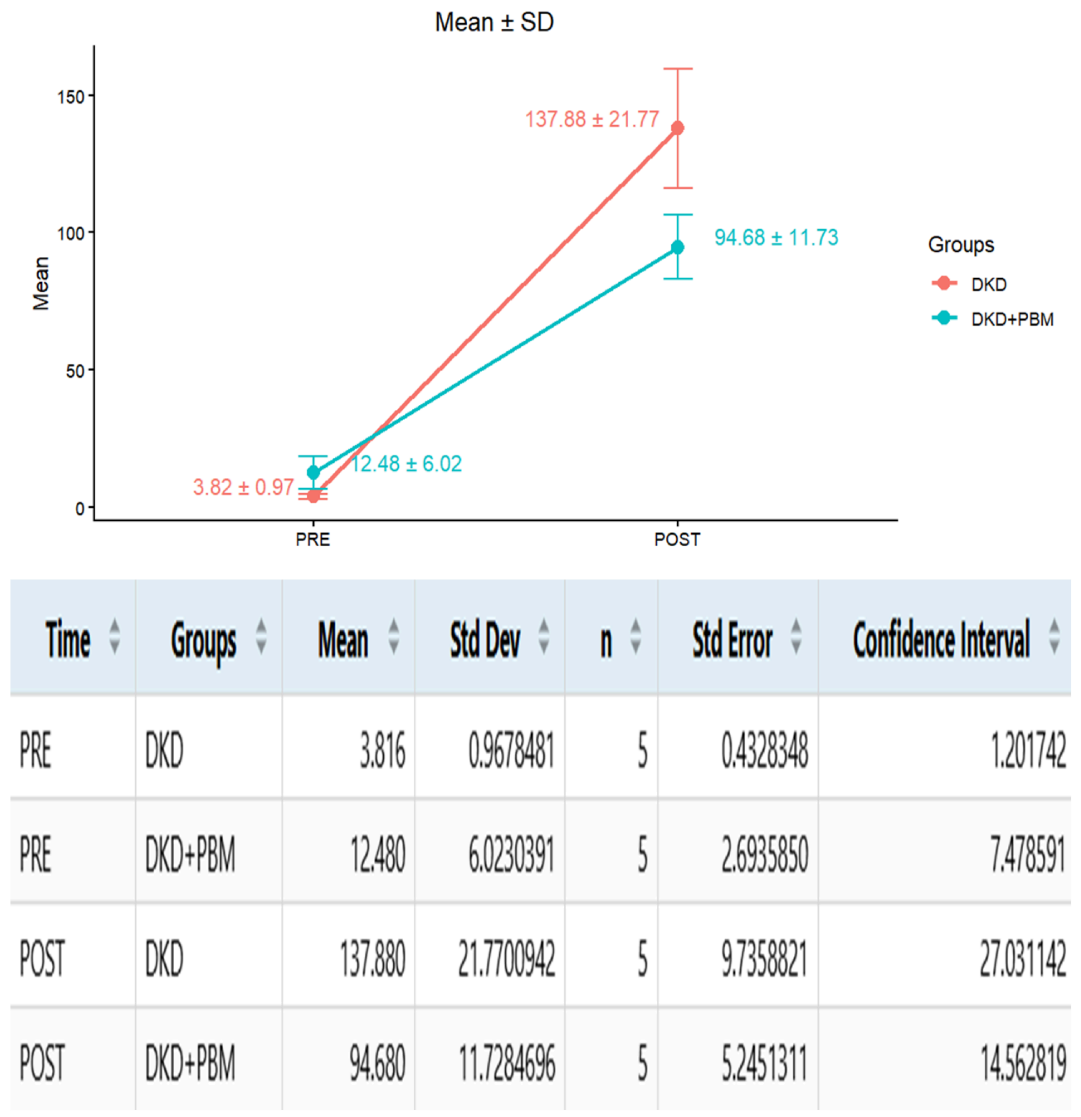
Evolution of albuminuria in DKD rats before and after diabetes induction. Albuminuria levels (mg/24 h) were assessed in the untreated DKD and photobiomodulation-treated DKD + PBM groups before (pre-induction) and after (post-induction) the establishment of diabetes. A significant increase in urinary albumin excretion was observed after induction in both groups, with attenuated values in the DKD + PBM group compared with the DKD group. Data are expressed as mean ± SD. Statistical analysis was performed using a mixed-effects model.

In the longitudinal analysis (Figure 4), both the DKD and DKD + PBM groups showed comparable baseline values during the pre-induction phase, confirming homogeneity prior to diabetes onset. Following induction, urinary albumin excretion increased significantly in both groups, consistent with glomerular injury characteristic of diabetic kidney disease. However, this increase was markedly attenuated in the DKD + PBM group, which displayed significantly lower values compared with the untreated DKD group. Collectively, these findings indicate that photobiomodulation exerted a partial renoprotective effect, mitigating the progression of albuminuria in diabetic rats and supporting a relative preservation of glomerular function in the context of hyperglycemia-induced injury.

**Figure 5:**
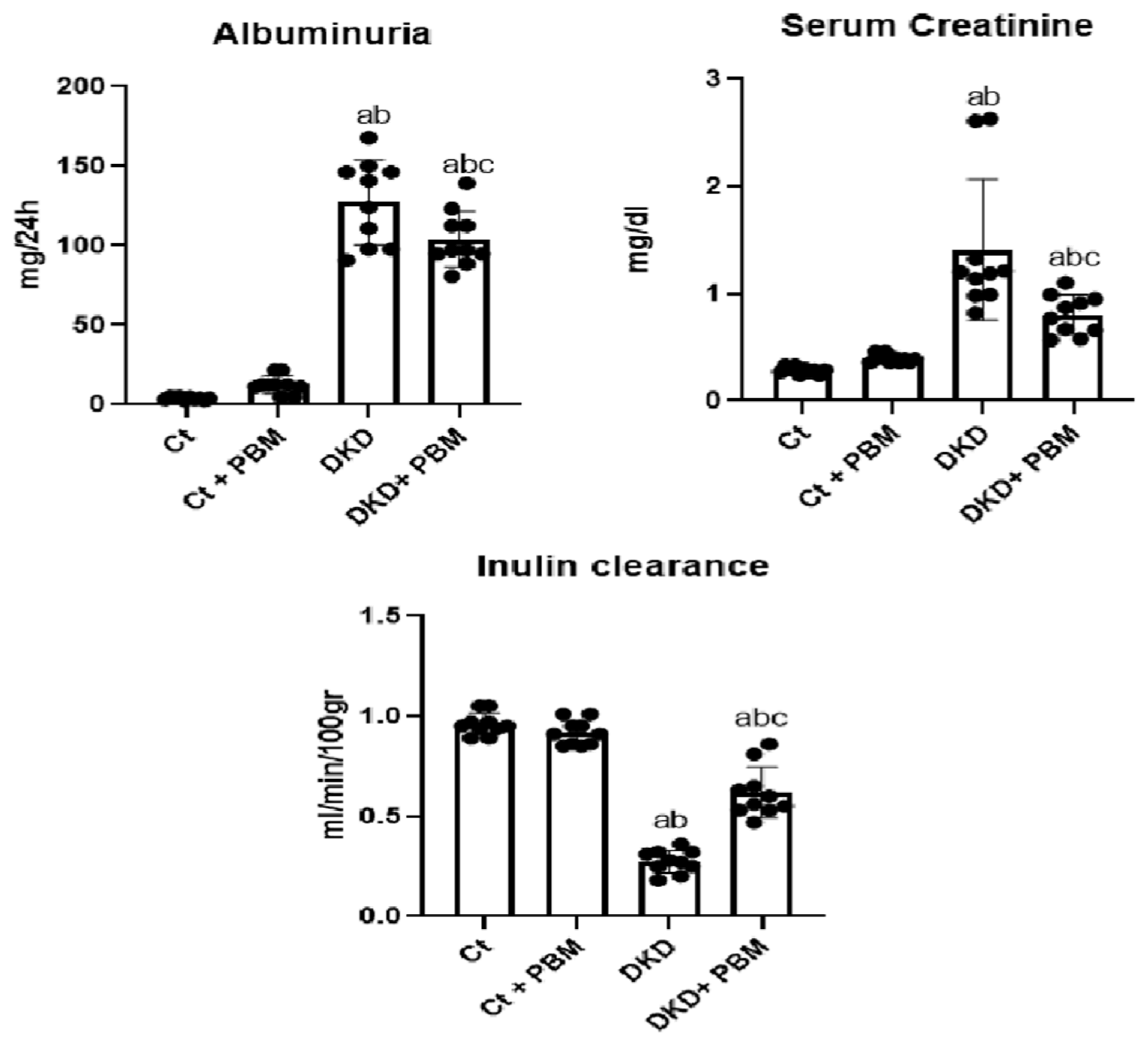
Kidney function parameters. Albuminuria (mg/24 h), (serum creatinine (mg/dL), and inulin clearance (mL/min/100 g), were evaluated at the end of the experimental protocol in Ct, Ct + PBM, DKD, and DKD + PBM groups. Data are expressed as mean ± SD. Statistical analysis was performed using one-way ANOVA, followed by Tukey’s post hoc test. Albuminuria: DKD differed significantly from Ct and Ct + PBM (*p* < 0.0001); DKD + PBM differed from DKD (mean difference = 23.19; 95% CI [3.612 to 42.77]; *p* = 0.0149), although it remained significantly elevated compared with controls. Serum creatinine: DKD differed significantly from Ct and Ct + PBM (*p* < 0.0001); DKD + PBM differed from DKD (mean difference = 0.6020; 95% CI [0.1917 to 1.012]; *p* = 0.0019), although it remained significantly elevated compared with controls (*p* = 0.0082 vs. Ct; *p* = 0.0480 vs. Ct + PBM). Inulin clearance: DKD and DKD + PBM differed significantly from Ct and Ct + PBM (*p* < 0.0001); DKD + PBM differed from DKD (mean difference = −0.3450; 95% CI [−0.4419 to −0.2481]; *p* < 0.0001), although it remained significantly lower than controls. Note: a—*p* < 0.05 vs. Ct; b—*p* < 0.05 vs. Ct + PBM; c—*p* < 0.05 vs. DKD.

In the evaluation of kidney function parameters (Figure 5), albuminuria levels were significantly elevated in the DKD groups compared with the Ct and Ct + PBM controls, indicating diabetes-induced glomerular impairment. Notably, the DKD + PBM group exhibited a significant reduction in albuminuria relative to the DKD group.

Serum creatinine analysis revealed no statistically significant differences between the Ct and Ct + PBM groups. In contrast, both DKD groups exhibited a significant increase in serum creatinine compared with controls, reflecting renal dysfunction associated with diabetes. The DKD + PBM group showed a significant reduction in serum creatinine levels compared with the DKD group, suggesting a potential protective effect of PBM on this parameter.

Regarding inulin clearance, a significant reduction was observed in the DKD and DKD + PBM groups compared with the Ct and Ct + PBM groups, indicating a decrease in glomerular filtration rate induced by the diabetic condition. On the other hand, the DKD + PBM group demonstrated a significant increase in inulin clearance compared with the DKD group, suggesting a potential renoprotective effect of PBM treatment.

### 2.4. Oxidative and Antioxidant Profile

**Figure 6:**
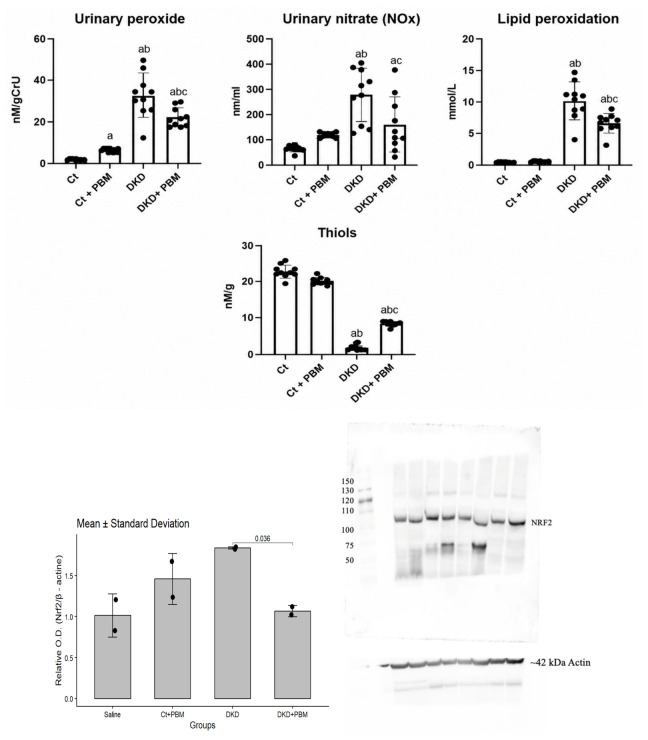
Oxidative and antioxidant profile. Urinary peroxide (nMol/g Cr-U), urinary nitrate (NOx, nM/mL), lipid peroxidation (mmol/L), thiol content (nM/g), and Nrf2 expression were evaluated in Ct, Ct + PBM, DKD, and DKD + PBM groups at the end of the experimental protocol. Data are expressed as mean ± SD. Statistical analysis was performed using one-way ANOVA, followed by Tukey’s post hoc test. Urinary peroxide: DKD differed significantly from Ct and Ct + PBM (*p* < 0.0001); DKD + PBM differed from DKD (mean difference = 10.54; 95% CI [3.501 to 17.57]; *p* = 0.0015), although it remained significantly elevated compared with controls. Urinary nitrate (NOx): DKD differed significantly from Ct and Ct + PBM (*p* < 0.0001); DKD + PBM differed significantly from DKD (mean difference = 117.9; 95% CI [25.97 to 209.9]; *p* = 0.0075) and from Ct (mean difference = −94.61; 95% CI [−186.6 to −2.648]; *p* = 0.0418), with no significant difference relative to Ct + PBM (*p* = 0.6481). Lipid peroxidation: DKD differed significantly from Ct and Ct + PBM (*p* < 0.0001); DKD + PBM differed from DKD (mean difference = 3.526; 95% CI [1.498 to 5.554]; *p* = 0.0002), although it remained significantly elevated compared with controls. Thiol content: All pairwise comparisons were statistically significant (*p* < 0.0001), including Ct + PBM vs. Ct (mean difference = 2.574; 95% CI [1.211 to 3.938]), indicating enhanced antioxidant capacity even under physiological conditions. DKD + PBM differed significantly from DKD (mean difference = −6.458; 95% CI [−7.822 to −5.094]; *p* < 0.0001), although levels remained significantly lower than controls. Western blotting of Nrf2: In kidney tissue, normalized to β-actin, groups (*n* = 2/group). Data are expressed as mean ± SD. A planned comparison (Welch’s *t*-test with Sidak correction) between DKD and DKD + PBM showed a significant reduction in Nrf2 expression with PBM treatment (mean difference = 0.770; 95% CI [0.210–1.330]; *p* = 0.036). Full, uncropped blot images are shown. Primary antibody: rabbit anti-Nrf2 (HL1021, Sigma-Aldrich/Merck, Darmstadt, Germany). Note: a—*p* < 0.05 vs. Ct; b—*p* < 0.05 vs. Ct + PBM; c—*p* < 0.05 vs. DKD.

In the analysis of oxidative metabolites (Figure 6), no statistically significant differences were observed between the control groups. In contrast, urinary peroxide levels were significantly increased in the DKD and DKD + PBM groups compared with controls, indicating enhanced oxidative stress associated with diabetes. Notably, the DKD + PBM group showed a significant reduction in this marker compared with the DKD group, suggesting a potential antioxidant effect of PBM treatment.

For urinary nitrate levels, no significant differences were observed between the control groups. The DKD groups displayed a marked increase compared with controls, reflecting enhanced production of reactive nitrogen species, a hallmark of hyperglycemia induced oxidative stress. In contrast, the DKD + PBM group did not differ significantly from the controls but showed a significant reduction relative to the DKD group, indicating a modulatory effect of PBM on nitrogen oxide production in diabetic animals.

Regarding lipid peroxidation, no differences were detected between the control groups. Both the DKD and DKD + PBM groups exhibited significantly elevated values compared with controls, reflecting increased oxidative stress induced by hyperglycemia. However, DKD + PBM animals demonstrated a significant reduction compared with the DKD group.

Renal redox status, assessed through soluble thiol levels, revealed a significant increase in the Ct + PBM group compared with the Ct group, suggesting enhanced antioxidant capacity even under physiological conditions. The animals in the DKD and DKD + PBM groups exhibited a significant reduction in thiol levels compared with the controls, reflecting the oxidative stress burden associated with diabetes. Importantly, the DKD + PBM group demonstrated a significant increase in thiols compared to the DKD group, indicating a restorative effect of PBM on redox balance in the diabetic condition.

Finally, analysis of the antioxidant regulator nuclear factor erythroid 2-related factor 2 (Nrf2) revealed a significant increase in the DKD group compared with Ct and Ct + PBM controls, consistent with the activation of compensatory antioxidant pathways in response to oxidative stress. In contrast, Nrf2 expression was significantly reduced in the DKD + PBM group compared with DKD, suggesting modulation of this pathway by PBM treatment.

### 2.5. Inflammatory Profile

**Figure 7:**
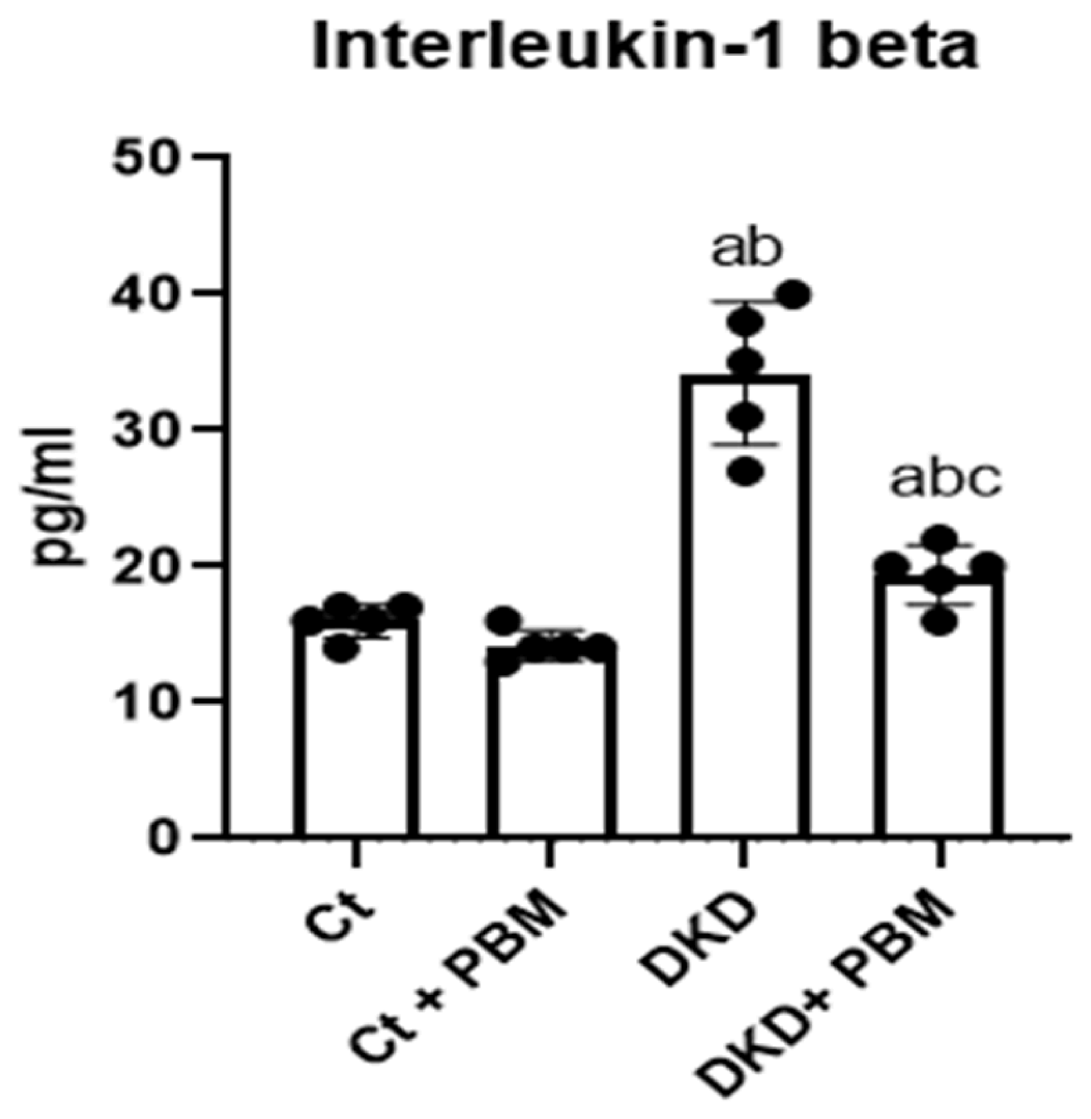
Interleukin-1β levels. Quantification of interleukin-1β (IL-1β), a pro-inflammatory cytokine associated with the early phase of the inflammatory response, was performed by ELISA in renal homogenates. Values were normalized to total protein concentration and expressed as pg/mg. Data are presented as mean ± SD. Statistical analysis was performed using one-way ANOVA, followed by Tukey’s post hoc test. No significant difference was found between Ct and Ct + PBM (mean difference = 1.8; 95% CI [−2.351 to 5.951]; *p* = 0.4027). The DKD group showed significantly elevated IL-1β compared with Ct (mean difference = −18.2; 95% CI [−29.15 to −7.246]; *p* = 0.0086) and Ct + PBM (mean difference = −20.0; 95% CI [−28.44 to −11.56]; *p* = 0.0023). PBM treatment led to a significant reduction in IL-1β in the DKD + PBM group compared with DKD (mean difference = 14.8; 95% CI [1.901 to 27.70]; *p* = 0.0318), although it remained significantly elevated relative to Ct (mean difference = −3.4; 95% CI [−5.476 to −1.324]; *p* = 0.009), with no significant difference relative to Ct + PBM (mean difference = −5.2; 95% CI [−11.16 to 0.7551]; *p* = 0.0763). Note: a—*p* < 0.05 vs. Ct; b—*p* < 0.05 vs. Ct + PBM; c—*p* < 0.05 vs. DKD.

In the analysis of renal IL-1β levels, no statistically significant differences were detected between the Ct and Ct + PBM groups (Figure 7). In contrast, both diabetic groups exhibited a marked increase in IL-1β concentrations compared with controls, indicating an exacerbated inflammatory response associated with diabetes. Notably, the DKD + PBM group showed a significant reduction in IL-1β levels compared to the DKD group, suggesting a potential anti-inflammatory effect of PBM treatment in the context of diabetic renal injury.

### 2.6. Histology

**Figure 8:**
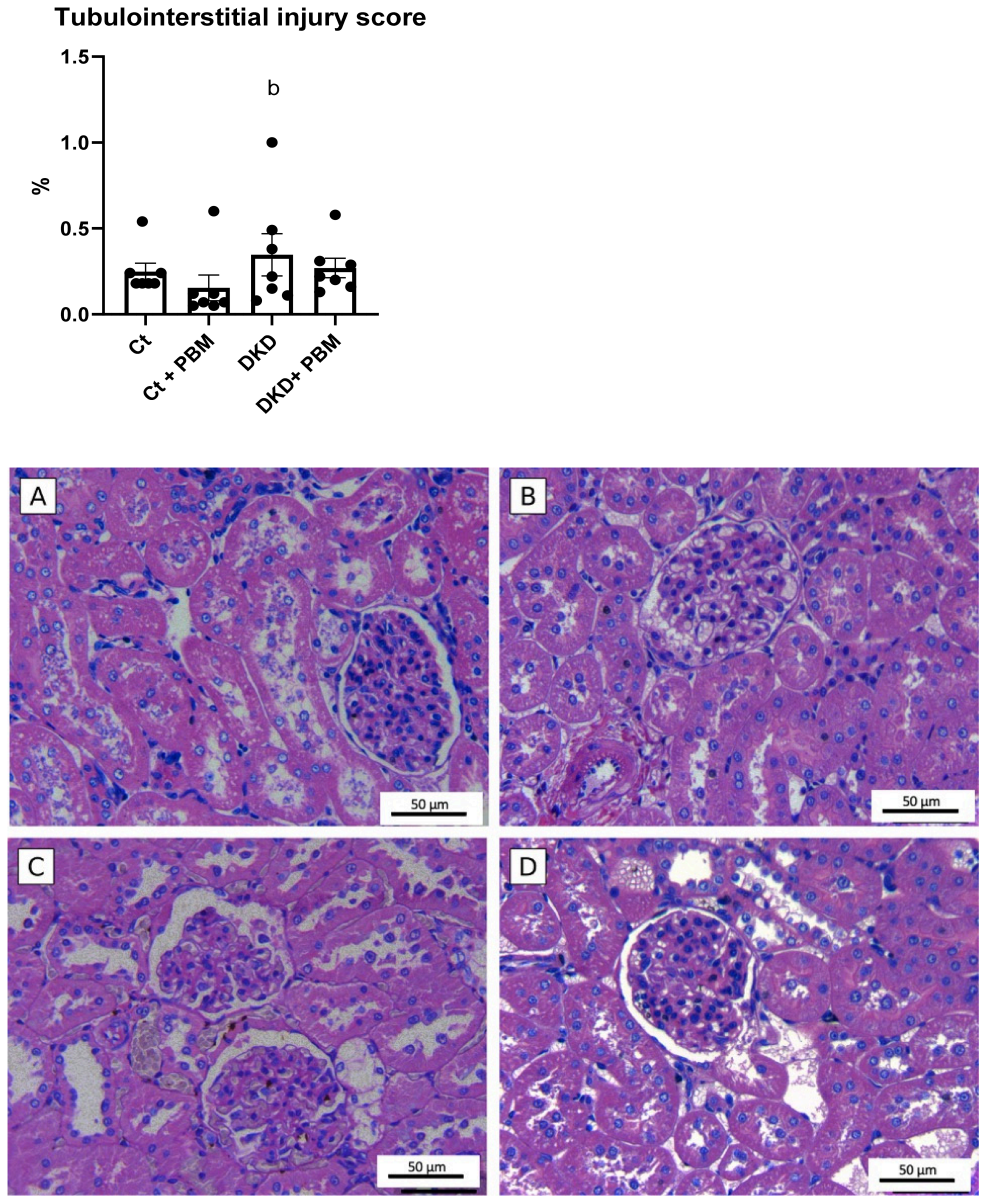
Tubulointerstitial injury score and photomicrographs of renal cortical tissue of the groups: (**A**) Ct, (**B**) Ct + PBM, (**C**) DKD, and (**D**) DKD + PBM. The bar graph represents the semiquantitative tubulo interstitial injury score (0–4), according to the percentage of cortical involvement. Data are expressed as mean ± SD. *p* < 0.05 compared to the control group. Note: b—*p* < 0.05 vs. Ct + PBM vs. DKD + PBM. Representative photomicrographs of renal cortical tissue stained with hematoxylin and eosin (400×) show tubular–interstitial changes in experimental groups. Arrows indicate epithelial cell flattening, tubular dilation, vacuolar degeneration, and inflammatory cell infiltration.

The histopathological analysis of the kidney tubulointerstitial compartment did not reveal statistically significant differences between the Ct and Ct + PBM groups, indicating preservation of renal morphology in non-diabetic animals (Figure 8). In the DKD groups, a significant increase in the tubulointerstitial lesion score was observed compared to the control groups, reflecting the impact of streptozotocin-induced diabetes. However, the comparison between the DKD and DKD + PBM groups did not show any statistically significant differences.

Taken together, the present findings suggest that PBM may confer renoprotection in experimental DKD across multiple domains. Relative to untreated DKD animals, PBM-treated diabetic rats showed improvements in glomerular filtration (inulin clearance), albuminuria, and serum creatinine, along with more favorable renal hemodynamics (mean arterial pressure, vascular resistance, and renal blood flow), attenuation of oxidative stress markers (urinary peroxides, lipid peroxidation, nitrate, and renal thiols), reduction in renal IL-1β concentration, and lower Nrf2 protein expression—findings consistent with a partial modulation of the oxidative and inflammatory state. Notably, the tubulointerstitial injury score did not differ significantly between the DKD and DKD + PBM groups. These observations are examined in the following sections.

## 3. Discussion

Overall, the findings of this study confirm the robustness of the STZ-induced type 1 diabetes model [10,11], as reflected by sustained hyperglycemia. Although the STZ-induced model reproduces type 1 insulin-deficient diabetes, it remains a well-established and mechanistically relevant model for investigating DKD, regardless of diabetes subtype. This is because renal injury in DKD is primarily driven by chronic hyperglycemia through glucotoxicity-mediated pathways, including increased polyol and hexosamine flux, DAG–PKC activation, advanced glycation end products, accumulation, oxidative stress, and NF-κB-mediated inflammation, which are activated once persistent hyperglycemia is established, irrespective of whether it results from insulin deficiency (type 1 diabetes) or insulin resistance (type 2 diabetes). As the present study specifically evaluated the effects of PBM on these glucotoxicity-driven mechanisms, the STZ model provides a biologically relevant platform for investigating the renoprotective effects of PBM with potential translational applicability to both type 1 and type 2 DKD. Consistent with previous reports, diabetic animals also exhibited the classical clinical manifestations of uncontrolled diabetes, including polyphagia, polydipsia, polyuria, and progressive weight loss. Importantly, photobiomodulation was associated with a significant reduction in blood glucose levels [12,13] and attenuation of catabolic symptoms, supporting the hypothesis that PBM exerts beneficial effects on glucose metabolism under conditions of chronic hyperglycemia [14]. Although PBM produced a significant reduction in blood glucose levels, diabetic animals remained markedly hyperglycemic throughout the experimental period, and normoglycemia was not restored. Therefore, the beneficial renal effects cannot be attributed solely to improved glycemic control. Chronic hyperglycemia, the principal driver of diabetic kidney injury, persisted throughout the study, indicating that the attenuation of renal dysfunction likely resulted from additional biological mechanisms beyond lowering glucose. This interpretation is consistent with previous evidence demonstrating that PBM exerts direct cytoprotective effects through the modulation of mitochondrial function, oxidative stress, inflammatory signaling, and tissue repair pathways independently of complete normalization of blood glucose. Thus, although partial improvement in glycemic status may have contributed to the observed response, it is unlikely to fully explain the magnitude of the renoprotective effects observed in the present study. These results are consistent with the observations of Gou et al. (2020) [13] and Gong et al. (2021) [12], who demonstrated that PBM in murine models of diabetes improved insulin sensitivity and preserved pancreatic β-cell function. Mechanistically, these effects are likely mediated through mitochondrial modulation, AMPK activation, and enhanced GLUT4 translocation to the cell membrane [15,16], promoting greater peripheral glucose uptake and thereby reducing the hypercatabolic state. Taken together, these data highlight PBM as a non-invasive, low-cost, and promising therapeutic approach with translational potential for the management of metabolic and renal complications associated with diabetes mellitus.

Moreover, in DKD animals, we observed higher albuminuria, increased serum creatinine (SCr), reduced renal blood flow (RBF), and increases in mean arterial pressure (MAP) and renal vascular resistance (RVR)—a pattern consistent with early glomerular hyperfiltration driven by afferent dilatation and efferent constriction, followed by functional decline [17,18]. Consistently, the glomerular filtration rate (GFR), measured by gold-standard inulin clearance, was reduced in the DKD groups, supporting the validity of the model for diabetic kidney disease under sustained hyperglycemia. In contrast, photobiomodulation (PBM) was associated with significant improvements in renal hemodynamics and function, with higher inulin-based GFR, increased RBF, and lower MAP, as well as RVR relative to untreated DKD. These findings accord with prior preclinical reports in hypertensive rats that showed PBM mediated reductions in systemic blood pressure and heart rate [19,20], and aligned with the synthesis provided by Bian et al. (2022), who reported renoprotective effects of PBM across experimental and clinical chronic kidney disease settings, including lower blood pressure, higher GFR, and reduced tubulointerstitial fibrosis [9]. Extending this mechanistic plausibility, Ucero et al. (2013) [21] demonstrated that PBM attenuates oxidative stress and inflammation in diabetic kidneys, while Hamblin (2017) [22] highlighted improvements in microcirculation, modulation of inflammatory responses, and stimulation of tissue repair—features that together offer a coherent framework for the hemodynamic and functional benefits observed here.

With respect to oxidative metabolites urinary peroxides, lipid peroxidation products, and urinary nitrate and antioxidant enzymes, DKD animals exhibited oxidative stress and redox imbalance. These findings are consistent with the effects of glucotoxicity on the activation and dysfunction of cellular metabolic pathways [17,18,23], culminating in mitochondrial dysfunction and facilitating inflammatory pathway activation [24,25]. In the DKD group treated with photobiomodulation, we observed a significant reduction in urinary peroxides and urinary nitrate. Mechanistically, this effect could partly be explained by the model proposed by Karu et al. (2008) [26], whereby light induces photodissociation of NO from cytochrome c oxidase (CCO), restoring mitochondrial electron flux and attenuating redox stress. This indicates that under normoxic conditions, PBM can mobilize NO from intracellular stores, increasing its bioavailability and promoting cytoprotective effects [26,27,28,29].

Conversely, in hypoxic microenvironments characteristic of diabetic nephropathy, PBM may stimulate endogenous NO synthesis via CCO, as demonstrated by Ball et al. (2011) [5], thereby favoring vasodilation, angiogenesis, and improved renal perfusion—benefits that reinforce the therapeutic potential of PBM in settings of endothelial dysfunction.

Regarding the observed increase in antioxidant enzyme expression and non-enzymatic thiols in the PBM-treated DKD group, the data suggest strengthening of antioxidant defenses and enhanced buffering capacity against reactive oxygen species (ROS), which are in line with the hypothesis of mitochondrial ROS as second messengers in redox-dependent signaling pathways [7,30,31]. Such signaling activates antioxidant transcriptional programs, including Nrf2, an effect also evidenced in our study, thereby supporting the renoprotective role of PBM [5,6].

Under physiological conditions, Nrf2 is constitutively bound to its cytosolic repressor Keap1, which targets it for ubiquitin-proteasomal degradation via the CUL3 ligase complex; in response to oxidative stress, reactive cysteine residues within Keap1 may be modified, potentially releasing Nrf2 to translocate to the nucleus and activating antioxidant response element (ARE)-driven cytoprotective genes, including those encoding superoxide dismutase, catalase, and heme oxygenase-1 (HO-1), as well as glutamate cysteine ligase, the rate-limiting enzyme in glutathione synthesis [32]. In the untreated DKD group, the elevated Nrf2 expression observed in the present study may reflect a compensatory upregulation in response to sustained hyperglycemia-driven oxidative stress, consistent with patterns reported in experimental renal disease models in which Nrf2 expression appears to increase during early disease progression before declining below baseline as injury advances [33]. The reduction in Nrf2 expression observed in DKD + PBM animals may therefore not represent pathway suppression per se but rather a potential feedback normalization consequent to the attenuation of the oxidative stimulus. Photobiomodulation has been reported to upregulate antioxidant defenses and reduce oxidative stress through the activation of downstream transcription factors [7], and recent evidence suggests that the anti-inflammatory effects of red light may operate through a Nrf2-dependent mechanism, as the silencing of Nrf2 by siRNA abolished the regulatory effect of light on cytokine production in keratinocytes challenged with a pro-inflammatory stimulus [34]. In this context, as PBM was associated with reduced urinary peroxides, lipid peroxidation, and nitrate levels, the redox signal sustaining Keap1 inactivation and Nrf2 nuclear accumulation may have been diminished, allowing Keap1-mediated proteasomal degradation to partially resume. This hypothesis is further supported by the parallel increase in renal thiol content observed in DKD + PBM animals. Soluble thiols, particularly reduced glutathione (GSH), constitute a primary non-enzymatic antioxidant buffer whose synthesis is regulated transcriptionally downstream of Nrf2/ARE activation [32]; higher thiol levels in DKD + PBM animals may suggest that, despite lower total Nrf2 protein expression, ARE-driven antioxidant programs had been sufficiently engaged to restore non-enzymatic redox buffering capacity, potentially rendering sustained Nrf2 elevation no longer necessary to maintain redox homeostasis. Taken together, lower Nrf2 expression accompanied by higher renal thiols in DKD + PBM animals may be more consistent with a restored redox balance in which antioxidant defenses appear adequate, and the compensatory transcriptional response has been more downregulated than with impaired cytoprotection; however, direct causal evidence in the DKD setting remains to be established.

Additionally, we observed a significant increase in interleukin-1 beta (IL-1β) in DKD animals, consistent with the role of advanced glycation end products (AGEs) [30,35] in NF-κB activation, downregulation of NO production, and promotion of ROS [3,36], contributing to glomerulosclerosis and podocyte injury. Chronic inflammation is sustained by persistent activation of pro-inflammatory pathways [4,25] and continuous release of cytokines, including IL-1β, TNF-α, and IL-6, which amplify tissue damage [24,37]. In contrast, PBM-treated DKD animals showed lower IL-1β compared to DKD, in agreement with Abidi et al. (2020) [38], who demonstrated modulation of inflammatory responses in human periodontal ligament fibroblasts irradiated at 810 nm.

Despite the protective effects of photobiomodulation observed with renal function, oxidative and inflammatory profiles, and renal hemodynamics in this experimental model, histological analysis of the tubulointerstitial injury score did not reveal statistically significant differences between the treated and untreated DKD groups.

This finding suggests that under the evaluated conditions, such as intervention duration, laser intensity, and disease stage, PBM was not sufficient to reverse or significantly attenuate the tubulointerstitial morphological alterations induced by diabetes mellitus. This absence of histological improvement does not diminish the biological relevance of the functional findings; instead of reflecting an isolated hemodynamic effect, the renoprotective actions of PBM were observed consistently across multiple pathophysiological domains, including renal function, renal hemodynamics, oxidative stress, and inflammatory parameters, consistent with a coordinated biological response. Nevertheless, the possibility that these early functional and molecular improvements do not necessarily translate into long-term structural protection cannot be excluded. This interpretation is consistent with the current understanding of diabetic kidney disease, in which functional, hemodynamic, inflammatory, and redox abnormalities precede the development and progression of structural kidney injury [3,37,39,40]. Likewise, PBM is thought to exert its primary biological effects through modulation of mitochondrial function, oxidative stress, inflammatory signaling, and microvascular perfusion [7]. Accordingly, the present findings are compatible with the possibility that the study conditions, including the duration of the intervention and the stage of disease evaluated, were sufficient to attenuate early pathogenic mechanisms without yet producing detectable structural remodeling. These results should therefore be interpreted as evidence of early functional and molecular renoprotection rather than as definitive structural renoprotection, and longer-term studies incorporating more comprehensive histopathological assessments, including fibrosis, glomerulosclerosis, and extracellular matrix deposition, will be necessary to determine whether these early biological effects ultimately translate into sustained structural preservation.

Taken together, these results could support multiple mechanistic hypotheses for the benefits of PBM, where the absorption of light by mitochondrial chromophores (such as CCO) triggers responses that extend beyond bioenergetics, influencing nuclear gene expression via MAPK, PI3K/Akt, and NF-κB [38], in addition to improving microcirculation and modulating inflammation [22,41]. In light of these principles, it is essential to recognize that the biological effects of PBM do not converge on a single molecular target but instead reflect a dynamic, adaptive, and microenvironment-dependent process. It should be emphasized that the present study was not intended to investigate these molecular mechanisms directly; therefore, the proposed pathways should be interpreted as biologically plausible hypotheses supported by previous experimental evidence rather than as mechanistic findings derived from the current investigation [22,29,41]. Accordingly, PBM acts as a physiological modulator [6,29], promoting biostimulation [6,42], cellular repair [8,39,43], and homeostatic re-equilibration [14,16,44] across distinct pathophysiological contexts. Recognizing this complexity underscores the importance of careful cellular state characterization and personalization of therapeutic parameters (wavelength, dose, irradiance, and temporal regimen) to maximize the clinical benefit from photobiomodulation.

Therefore, although the present findings are promising and support the therapeutic potential of photobiomodulation as an adjuvant strategy in diabetic kidney disease, the consolidation of its clinical relevance necessarily depends on the translation of these results into well-designed controlled clinical trials in humans. Such studies will be essential to validate its efficacy and safety, as well as to establish optimal treatment parameters and determine its real impact on disease progression.

In conclusion, this study expands the current preclinical evidence supporting PBM as a therapeutic modulator in DKD and provides a robust foundation for future clinical investigations. The transition from experimental models to clinical application represents a critical step toward positioning photobiomodulation as a viable and integrative therapeutic approach in the management of diabetic kidney disease.

## 4. Materials and Methods

### 4.1. Animal Model and Ethical Approval

Male Wistar rats, weighing between 250 and 290 gr, were used. Only male animals were used in order to avoid the hormonal variability related to the estrous cycle, which is well-documented in female rodents and can influence oxidative stress, inflammatory, and hemodynamic parameters, thereby ensuring greater homogeneity and reproducibility of the evaluated outcomes. Future studies by our group will extend this protocol to female rats once the methodology has been further standardized in our laboratory, allowing for sex-based comparative analysis. The animals were kept with free access to water and chow, and housed in collective cages under controlled temperature conditions with alternating light and dark cycles in the Animal Model Experimental Laboratory of the School of Nursing, University of São Paulo.

All procedures performed in this study were carried out in accordance with the ethical principles for animal experimentation adopted by the Brazilian College of Animal Experimentation (COBEA) and the Ethics Committee on the Use of Animals of the University of São Paulo Medical School (CEUA—FMUSP) [45], and was approved under protocol no. 2036/2023.

### 4.2. Experimental Design and Grouping

Sample size was determined based on previous studies in renal models, ensuring adequate statistical power to detect biologically relevant differences. Each experimental group consisted of 10 animals (n = 10). Animals were randomly allocated to four experimental groups using a computer-generated randomization sequence Microsoft Excel, RAND function. Group allocation was performed by an independent investigator not involved in outcome assessment to minimize selection bias. The groups were defined as follows: Ct, n = 10; animals that received citrate buffer (diluent of streptozotocin—STZ; pH 4.2; i.v.; single dose; on the 1st day of the experimental protocol). Ct + PBM, n = 10; citrate animals that, starting on the third day of the protocol, were subjected to the photobiomodulation procedure (irradiation three times per week, for six weeks, wavelength of 808 nm, power of 100 mW, energy of 3 Joules, fluence of 30.48 J/cm^2^). DKD, n = 10; animals that received streptozotocin (STZ, 60 mg/kg; i.v.; single dose; diluted in 0.1 M citrate buffer; 1st day of the experimental protocol). DKD + PBM, n = 10; DKD animals that, starting on the third day of the protocol, were subjected to PBM as described above.

The experimental protocol lasted 6 weeks (45 days), during which the animals’ body weight and blood glucose levels were monitored weekly. On day 44, the animals were placed in metabolic cages for 24 h to measure urine volume and water and food intake. The urine samples collected were used for renal function and oxidative stress analyses. On day 45, the animals were anesthetized with xylazine (10 mg/kg) and ketamine (90 mg/kg) administered intraperitoneally for the evaluation of renal function by inulin clearance, followed by laparotomy and terminal blood collection via puncture of the abdominal aorta. The right kidney was removed and weighed to calculate the kidney weight-to-body weight ratio, while the left kidney was stored at −80 °C for subsequent biochemical analyses [46].

### 4.3. Induction of Diabetes

Type 1 diabetes mellitus was induced by a single intravenous (caudal vein) injection of streptozotocin (STZ; 60 mg/kg; Sigma Chemical Company, St. Louis, MO, USA) freshly dissolved in 0.1 M citrate buffer (pH 4.2). Animals with fasting blood glucose levels > 250 mg/dL, measured 48 h after injection, were considered diabetic. Blood glucose was determined from fresh capillary blood obtained by a tail prick using test strips and a glucometer (Accu-Chek^®^ Active, Roche Diagnostics, Seattle, WA, USA). Body weight and blood glucose were monitored weekly for the following four weeks [47].

### 4.4. Photobiomodulation Protocol

Photobiomodulation (PBM) was initiated on the third day of the experimental protocol, 48 h after the induction of type 1 diabetes mellitus. The procedure consisted of transcutaneous irradiation targeted to the renal region, using an infrared wavelength of 808 nm with an output power of 100 mW. An energy of 3 Joules was delivered per each irradiation point, corresponding to a fluence of 30.48 J/cm^2^ per point. Bilateral irradiation was performed, with one point applied over each kidney (right and left flanks), resulting in a total delivered energy of 6 Joules per session. PBM was applied three times per week for six consecutive weeks, totaling 18 sessions (Table 1). The protocol was adapted from a previous study by Conceição Filho, with adjustments for renal targeting in a diabetic kidney disease model [19].

The study by Conceição Filho was used as the initial reference for defining the irradiation schedule number of sessions and the energy applied, with the 808 nm infrared wavelength selected as it lay within the optical window of greatest tissue penetration and was suitable for transcutaneously reaching the kidneys. Based on this reference, two pilot tests were conducted to define the final protocol parameters, each performed with 3 animals per group. In the first test, 3 irradiation points were applied to each flank, with an energy of 6 Joules per point three times a week, using the dose employed by Conceição Filho as a reference. However, no statistically significant differences were observed in the parameters evaluated (capillary glycemia, weekly weight, and kidney function). This finding is consistent with previous reports, indicating that the biological effects of photobiomodulation follow a biphasic dose–response relationship, whereby higher doses may exert an inhibitory rather than a stimulatory effect [7]. Based on this rationale, in the second pilot test, the applied dose was reduced, using 1 irradiation point per flank, with an energy of 3 Joules per point three times a week. This latter protocol was selected, as it was this regimen in which a statistically significant difference was observed in the evaluated parameters; it was therefore adopted as the definitive photobiomodulation protocol used in the present study.

Laser application was performed in direct contact with previously shaved skin. The kidneys were identified by palpation of the abdominal region, and irradiation was delivered at a 90° angle to the skin surface over the right and left flanks to ensure consistent targeting.

The device used was a wireless therapeutic laser (Therapy EC^®^, DMC Equipamentos, São Carlos, SP, Brazil; ANVISA registration no. 80030819013), equipped with two laser diodes emitting red (660 nm) and infrared (808 nm) wavelengths. The infrared source consisted of an aluminum gallium arsenide (AlGaAs) semiconductor diode operating at a power of 100 mW in continuous mode.

### 4.5. Physiological and Biochemical Assessments

Physiological parameters included body weight, measured weekly, and capillary blood glucose, determined 24 h after the start of the experimental protocols and subsequently monitored weekly on days 2, 8, 15, 22, 29, 36, and 45. Blood glucose measurements were obtained from fresh capillary blood collected by tail pricks, using test strips and a glucometer (Accu-Chek^®^ Active, Roche Diagnostics).

Renal hemodynamics were evaluated by measuring mean arterial pressure (MAP) via carotid artery cannulation connected to a pressure transducer, and renal blood flow (RBF) using a perivascular ultrasonic flow probe placed around the left renal artery [10].

Kidney function was assessed by inulin clearance, as calculated from plasma and urine inulin concentrations during steady-state infusion. The jugular vein was catheterized (PE 60) for inulin infusion, and the carotid artery (PE 60) for blood sampling and hemodynamic monitoring. After a priming dose (100 mg/kg, i.v.) and continuous infusion (10 mg/kg; 0.04 mL/min) for two hours, urine was collected at 30 min intervals via bladder catheterization (PE 240) and blood samples at 60 min intervals. Plasma and urinary inulin concentrations were determined by the antrone colorimetric method (absorbance at 620 nm; spectrophotometer), and GFR, expressed as mL/min/100 g body weight [48] by measuring serum creatinine, was determined by the Jaffé colorimetric method. Urine samples were diluted (1:100 in distilled water), and then 1 mL of 0.75 N sodium hydroxide and 1 mL of 0.036 M picric acid were added. After homogenization and incubation for 20 min at room temperature, absorbance was read at 520 nm by spectrophotometry. Results were expressed as mg/dL [49]. Urinary albumin excretion was quantified by enzyme-linked immunosorbent assay (ELISA) using a commercial rat albumin kit (Rat Albumin ELISA Kit, Bethyl Laboratories Inc., Montgomery, TX, USA), following the manufacturer’s protocol. Results were expressed as ng/mL. The kidney weight-to-body weight ratio was calculated post-mortem.

The oxidative profile was determined by quantifying urinary hydrogen peroxide, and was quantified by the ferric-xylenol orange method (FOX-2). Briefly, 100 µL of urine was added to 900 µL of FOX-2 solution (90 mL methanol, 10 mL bidistilled water, 100 µM xylenol orange, 4 mM BHT, 25 mM H_2_SO_4_, and 250 µM ammonium ferrous sulfate; Vetec Química, RJ, Brazil), homogenized, and incubated for 30 min at room temperature. After centrifugation to remove protein residues, absorbance was read at 560 nm by spectrophotometry. Results were normalized to urinary creatinine and expressed as nmol/g [50]. Nitrate/nitrite (NOx) synthesis was assessed by urinary nitrite (NO_2_^−^) quantification using the Griess colorimetric method. Briefly, 150 µL of urine was added to 150 µL of Griess reagent (sulfanilic acid and alpha-naphthylamine hydrochloride in acidic medium, pH 2.5–5.0) and incubated for 15 min at room temperature. Absorbance was read at 545 nm using an ELISA microplate reader and compared against a sodium nitrite (NaNO_2_) standard curve (0.1–1.0 M). Results were normalized to urinary creatinine and expressed as nmol NO/g creatinine [51]. Lipid peroxidation was assessed by urinary malondialdehyde (MDA) quantification using the thiobarbituric acid reactive substances (TBARS) method. Briefly, 0.4 mL of urine was diluted with 0.6 mL of distilled water, followed by addition of 1.0 mL of 17.5% TCA and 1.0 mL of thiobarbituric acid (0.6%, pH 2), and then kept on ice. After homogenization, samples were heated in a boiling water bath for 20 min, cooled on ice, and 1.0 mL of 70% TCA was added. Following incubation for 20 min, samples were centrifuged at 3000 rpm for 15 min and absorbance read at 534 nm by spectrophotometry. MDA concentration was calculated using a molar extinction coefficient of 1.56 × 10^5^ M^−1^ cm^−1^ and expressed as nmol TBARS/g urinary creatinine [52]. Thiol groups were determined in renal tissue homogenates as a marker of oxidative stress. Tissue samples were homogenized in 10 mM sodium acetate buffer (0.5% Tween-20, DTPA, pH 6.5) and centrifuged at 5000 rpm for 10 min at 4 °C. The supernatant was deproteinized with 10% trichloroacetic acid (1:1) and recentrifuged. An aliquot of 400 µL was reacted with DTNB in Tris buffer (pH 8.0) for 10 min, and absorbance read at 412 nm by spectrophotometry. Total protein was quantified by the Bradford method (Bio-Rad kit, Hercules, CA, USA). Results were expressed as nmol thiols/mg total protein [53,54]. Antioxidant defense was assessed by measuring the protein expression of nuclear factor erythroid 2–related factor 2 (NRF2) via Western blotting. Renal lysates were prepared in RIPA buffer (50 mM Tris-HCl pH 7.5, 150 mM NaCl, 2 mM EDTA, 1% NP-40, 1% protease inhibitor cocktail; Sigma-Aldrich) and centrifuged at 12,000 rpm for 15 min at 4 °C. Proteins were separated by SDS-PAGE on 4–12% precast gels (Thermo Fisher Scientific, Singapore) and transferred onto PVDF membranes (Millipore, Burlington, MA, USA). Membranes were blocked with 5% BSA in TBS-T and incubated overnight at 4 °C with rabbit anti-Nrf2 primary antibody (HL1021, Sigma-Aldrich/Merck, Darmstadt, Germany; expected MW: ~95–110 kDa), followed by HRP-conjugated secondary antibody (Dako) for 1 h at room temperature. Bands were visualized by enhanced chemiluminescence (ECL), with β-actin as the loading control. Densitometric analysis was performed using ImageJ/Fiji 1.46 software (NIH, Bethesda, MD, USA) [55]. Inflammatory status was evaluated by quantifying interleukin-1β (IL-1β) in cortex and outer medulla homogenates, which were prepared in lysis buffer (50 mM Tris-HCl pH 7.4, 150 mM NaCl, 1% Triton X-100, 0.1% SDS, 1 µg/mL aprotinin, 1 µg/mL leupeptin, 1 mM PMSF, 1 mM sodium orthovanadate, 1 mM sodium pyrophosphate, 25 mM NaF, 0.001 M EDTA pH 8) at 4 °C and centrifuged at 10,000 rpm for 15 min. IL-1β was quantified in the supernatant using a commercial ELISA kit (R&D Systems Inc., Minneapolis, MN, USA) following the manufacturer’s instructions. Total protein was determined by the Bradford method and results expressed as pg/mg protein [56].

Finally, kidney histopathological analysis was performed on paraffin-embedded sections stained with hematoxylin and eosin, and tubular–interstitial lesions were scored on a semiquantitative scale (0–4) according to the extent of cortical involvement.

### 4.6. Data Analysis

Results were expressed as mean ± standard deviation. At the end of the study, the final data were analyzed using analysis of variance (ANOVA), followed by Tukey’s multiple comparison test. ANOVA tested the null hypothesis that the means of the four experimental groups (Ct, Ct + PBM, DKD, and DKD + PBM) were equal through the ratio between the variance explained by treatment (between groups) and the residual variance (within groups), expressed as the F statistic, along with its corresponding *p*-value and the coefficient of determination (R^2^), which indicates the proportion of total data variability explained by the experimental factor. Prior to ANOVA, the homogeneity of variances between groups was assessed using the Brown–Forsythe and Bartlett tests, which are both used to verify whether group standard deviations differ significantly from one another— an assumption relevant to the validity of the ANOVA model. When ANOVA indicated a significant difference between the means (*p* < 0.05), Tukey’s multiple comparisons test was applied, performing pairwise comparisons between all groups and providing the mean differences and corresponding 95% confidence intervals (95% CI) for each comparison, along with an adjusted *p*-values to control for the type I error rate arising from multiple simultaneous comparisons. No data points were excluded from the analysis; all animals within each experimental group were included in the statistical evaluation. For Nrf2 expression, given the pre-specified hypothesis of a treatment effect between the DKD and DKD + PBM groups, a planned comparison with Sidak correction was applied instead, providing the mean difference, 95% CI, and adjusted *p*-value for this single comparison. Statistical analyses were performed using Prism 8.0.

## 5. Conclusions

Photobiomodulation was associated with renoprotection in experimental diabetes, with an improvement in GFR (inulin clearance), with a reduction in albuminuria, and with better renal hemodynamics (increased renal blood flow, decreased mean arterial pressure, and decreased renal vascular resistance). These gains coincided with lower IL-1β and a redox shift consistent with reduced oxidative/inflammatory stress, alongside lower Nrf2 expression. Although mechanisms remain inferential and the study is limited by a single model, fixed parameters, and short follow-up, the data suggest PBM as a non-invasive, low-cost adjunct; however, its clinical utility remains to be confirmed in human clinical trials before parameter standardization and translational application can be considered.

## Figures and Tables

**Table 1:** Photobiomodulation parameters.

Parameter	Value
Wavelength	808 nm
Power output	100 mW
Mode	Continuous
Energy per point	3 J
Fluence per point	30.48 J/cm^2^
Number of points	2 (bilateral kidneys)
Total energy per session	6 J
Application	Transcutaneous, contact mode
Sessions per week	3
Duration	6 weeks (18 sessions)

## Data Availability

The data presented in this study are available on request from the corresponding author.

## Abbreviations

The following abbreviations are used in this manuscript:

AGEs	Advanced Glycation End Products
CCO	Cytochrome c Oxidase
DAG	Diacylglycerol
DM	Diabetes Mellitus
CKD	Chronic Kidney Disease
DKD	Diabetic Kidney Disease
ECL	Enhanced Chemiluminescence
OS	Oxidative Stress
PBM	Photobiomodulation
RBF	Renal Blood Flow
GSH	Reduced Glutathione
SAH	Systemic Arterial Hypertension
MDA	Malondialdehyde
NO	Nitric Oxide
MAP	Mean Arterial Pressure
PKC	Protein Kinase C
PVDF	Polyvinylidene Difluoride
RAGE	Receptors for Advanced Glycation End Product
RNS	Reactive Nitrogen Species
ROS	Reactive Oxygen Species
RVR	Renal Vascular Resistance
SCr	Serum Creatinine
SOD	Superoxide Dismutase
GFR	Glomerular Filtration Rate
RRT	Renal Replacement Therapy
VEGF	Vascular Endothelial Growth Factor
